# Co-infection of *Chlamydia psittaci* with H9N2, ORT and *Aspergillus fumigatus* contributes to severe pneumonia and high mortality in SPF chickens

**DOI:** 10.1038/s41598-017-14519-1

**Published:** 2017-10-25

**Authors:** Jun Chu, Qiang Zhang, Zonghui Zuo, Saeed El-Ashram, Yongxia Guo, Peng Zhao, Shujian Huang, Cheng He, Ahrar Khan

**Affiliations:** 1 College of Life Science and Engineering, Foshan University, Foshan, 528231 Guangdong, China; 2 Key Lab of Animal Epidemiology and Zoonosis of the Ministry of Agriculture, China. College of Veterinary Medicine, China Agricultural University, Beijing, 100193 China; 3 Faculty of Science, Kafrelsheikh University, Kafr El-Shiekh, 33735 Egypt; 4 Department of Pathology,Faculty of Veterinary Science, University of Agriculture, Faisalabad, 38040 Pakistan

## Abstract

Since 2007, most areas of China have seen outbreaks of poultry airsacculitis, which causes hugely economic losses to the poultry industry. However, there are no effective measures to combat the problem. In this study, 105 rations were collected to isolate *Aspergillus* spp. from the diseased farms. In subsequent experiments, SPF chickens were inoculated with *Ornithobacterium rhinotracheale* (ORT), *Chlamydia psittaci* (*C. psittaci*) and *Aspergillus fumigatus* (*A. fumigatus*), and mortality rate, body weight gain and lesion score were evaluated. Of these ration samples, 63 (60.0%) were *A. fumigates*, 21 (20.0%) were *Aspergillus niger* (*A. niger*) and 11 (10.5%) were *Aspergillus candidus* (*A. candidus*). Furthermore, SPF birds infected with *C. psittaci*, ORT, H9N2 virus and *A. fumigatus* conidia exhibited a mortality rate of 40%, while simultaneous co-infection with *C. psittaci*, ORT and *A. fumigatus* resulted in a mortality rate of 20%. The avian airsacculitis was manifested in the *C*. *psittaci* + ORT/*A. fumigatus*, *C. psittaci* + H9N2 + ORT/*A. fumigatus* and *C. psittaci* + H9N2/*A. fumigatus* groups while others had transient respiratory diseases without mortality. Our survey indicates that feed-borne *A. fumigatus* is prevalent in poultry rations. The combination of *C. psittaci*, ORT, H9N2 and *A. fumigatus* conidia contributes to the replication of avian airsacculitis by aggravating the severe damage to the air sacs and lungs of chickens.

## Introduction

Several infectious agents have been contributed to avian airsacculitis, such as *Aspergillus fumigatus* (*A. fumigatus*), *Ornithobacterium rhinotracheale* (ORT), avian influenza virus subtype H9N2 (H9N2 AIV)^1^, Newcastle disease virus (NDV), Infectious bronchitis virus (IBV), avian metapneumovirus (aMPV) and *Chlamydia psittaci* (*C.psittaci*)^2^. More recently, avian respiratory diseases of unknown etiology have become more prevalent in broilers and young laying hens in Northern China. Avian airsacculitis manifests itself on the first day after hatching and lasts for more than 30 days, resulting in 30% mortality rate in broiler farms during the winter seasons^3^. On the basis of condemnations alone, airsacculitis causes a tremendous economic loss in the poultry industry in China^4^.

In previous reports, ORT and *C. psittaci* have been identified in Belgian turkeys^5^. ORT and *Enterococcus faecalis* co-infection in chickens may be associated with the outbreak of chicken hemorrhagic pneumonia^6^. These pathogens should be considered when developing prevention strategies for respiratory disease in broilers^7,8^. Moreover, high seroprevalence of *C. psittaci* has been determined in Belgium, France and China with seropositive rates of 96%, 90% and 77.8%, respectively in broiler flocks^9–11^. A virulent *C. psittaci* strain has been isolated from meat pigeon farms with an outbreak involving severe respiratory signs^11^. Importantly, *C. psittaci* is also a zoonotic pathogen in humans and poses a potentially serious human health hazard^12^.

In addition to *C. psittaci* infection, H9N2 AIV has been associated with respiratory distress in birds^13^. It has also demonstrated that the co-infection of *C. psittaci* and H9N2 AIV induces 35% mortality rate with severe pneumonia in SPF chickens^14^. H9N2 AIV has been circulating world-wide in multiple avian species and humans^15^. In a pilot study, ORT, H9N2 and *Streptococcus zooepidemicus* have been isolated from commercial birds^3,16^. In the broiler industry, it is very difficult to select a vaccine strain that can work as a potent vaccine in the face of widely circulating mutants of an avian influenza virus^17^. Failure of *Mycoplasma Galliscepticum* (MG), IBV immunization strategy, outbreaks of avian airsacculitis are still a huge threat to chicken health, although the inactivated H9N2 vaccine, attenuated MG vaccine and live vaccine against IBV have been implemented^3,14^.

In a recent case, *A. fumigatus* has been isolated and identified from broiler grower ration that is above the nutrient standards during an outbreak of avian airsacculitis in Northern China^18,19^. In addition, an aflatoxin-induced immunosuppression has been reported in concurrent infection with H9N2 avian influenza virus^20^. However, co-infection with four pathogens and their potential role in airsacculitis have not been recorded. Our current experiment in a specific pathogen free (SPF) chicken model constitutes an exploration into the pathogenesis of avian airsacculitis and disease control strategies in poultry industry.

## Materials and Methods

### Determination of *Aspergillus* spp. in poultry feeds

Broiler feeds were collected from poultry farms suffered with severe diarrhea during 2015 and 2016, including starter (34), grower (36) and finisher (35) feed formulations. The samples were sent to the laboratory at China Agricultural University, China and examined upon arrival. Five-hundred grams of each sample were collected from different locations within the same diet and then mixed thoroughly for the inoculation of Sabouraud dextrose agar (SDA) medium, which contains chloramphenicol (0.5%) (Oxoid, Beijing, China)^21^. Fumonisin B1, B2 and B3 were subsequently determined using UPLC/ESI-MS/MS as previously described^22,23^.

### Identification of *Aspergillus fumigatus* by conventional PCR

The amplification was performed using specific primers^24^. All oligonucleotides used in this study were designed based on the relevant sequences of the internal transcribed spacer 1 (ITS1) ribosomal DNA. The primers were expected to amplify a fragment 521 bp in length, including the whole ITS1 region. Moreover, three oligonucleotide primers based on specific regions of ITS1 were designed for identifying *A. fumigatus*, *A. flavus* and *A. niger*. The *A. fumigatus* specific primers were made up of primers designated ASPU (5′-ACTACCGATTGAATGGCTCG-3′) and Af3r (5′-CATACTTTCAGAACAGCGTTCA-3′). The *A. niger* set was composed of ASPU and Ni1r (5′-ACGCTTTCAGACAGTGTTCG-3′), and the *A. flavus* set consisted of ASPU and Fl2r (5′-TTCACTAGATCAGACAGAGT-3′). However, the *A. candidus* specific primers were  designed according to the sequence of the 10-deacetylbaccatin III 10-O-acetyltransferase (dbat) gene (NCBI accession number EU883596.3). The designed primers were 5′-GAAGCCGGAAGACCCTTTATAC-3′ and 5′- CCCACCCAAAGTCTACTTCATC-3′. The PCR was carried out in a final volume of 25 μl containing 12.5 μl 2 × Taq PCR MasterMix (Aidlab Biotechnologies Co., Ltd,Beijing,China), 2 μl DNA template, 0.2 μl of each primer, and ultra-pure water was added up to 25 μl. PCR conditions were as follows: one pre-denaturation cycle at 94 °C for 5 min, 25 cycles of denaturation at 94 °C for 1 min, annealing at 60 °C for 15 s, elongation at 72 °C for 15 s and one post-elongation cycle at 72 °C for 10 min. PCR products were visualized in 1.20% agarose gel stained with ethidium bromide under UV transillumination.

### Animals and ethics statement

Eighty 10-day-old specific pathogen free (SPF) birds were procured from Beijing Merial Vital Bridge Laboratory Animal Co., Ltd and kept in negative-pressure isolators at China Agricultural University (Beijing, China). All animals were maintained, inoculated and euthanized according to the protocol approved by an ethical board at China Agricultural University on Institutional Animal Care and Use Committee (IACUC). This protocol follows humane protocols that minimize pain in the animals. Briefly, the birds were observed 2 or 3 times per day for signs, such as severe depression (ruffled feathers and reluctance to move, not moving when prodded and/or severe respiratory distress) or extreme injuries unrelated to treatment. Animals displaying these symptoms were removed and euthanized. No animals were died of influenza or unrelated injuries for the entire duration of the study. All animals were euthanized at the end of study in a CO_2_ chamber using 100% CO_2_ at a flow rate of 10–30% of the Chamber Volume per Minute (CV/min). The animals were observed for the absence of breathing and lack of the heartbeat. The CO_2_ flow was maintained for at least 1 min after respiratory arrest. After confirmation of death, an additional secondary physical euthanasia (i.e. cervical dislocation) was performed before tissue collection and carcass disposal.

### Isolation of pathogens

A highly virulent HJ strain of *C. psittaci* was isolated from diseased pigeons^11^ while avian influenza virus (H9N2/chicken/Shandong/2011)^3^ and *Ornithobacterium rhinotracheale* (ORT/chicken/Shandong/2011)^3^ were isolated from the broilers with hemorrhagic pneumonia.

### Experimental infection of SPF chickens with *C. psittaci*, H9N2, ORT and *A. fumigatus* conidia

Specific pathogen free (SPF) White Leghorn chickens were randomly divided into 8 groups (10 birds per group). At the age of three weeks, group 1 animals were received simultaneous intratracheal inoculation (it) of 1 × 10^8.5^ IFU *C*. *psittaci*, 1 × 10^8.0^ CFU ORT and intranasal administration (in) of 1 × 10^8.0^ EID_50_ H9N2, and then inoculated with 1 × 10^8.0^ CFU of the fungal spores per os (po). A second group of birds were inoculated with *C. psittaci* (it), H9N2 (in) and fungal conidia (po). Group 3 birds were given *C. psittaci* (it), ORT (in), and fungal spores (po) while group 4 birds were administered *C. psittaci* (it) and *A. fumigatus* conidia (po). Similarly, group 5 birds were inoculated with H9N2 (in) and then fed fungal spores (po), group 6 birds were administered ORT (in) and fungal conidia (po) and group 7 birds were only received *A. fumigatus* conidia (po). SPF chickens from group 8 were orally given physiological saline and served as the control group. All birds were observed daily, and the immune organ indices were determined at 5 and 10 days post-inoculation.

### Lesion scores of air-sacs and lungs

Air-sac and lung lesion scoring (Table 1 and Table 2) were determined as previously recorded^25,26^.

**Table 1:** Air sac lesion scoring in chickens.

Assigned score	Air sac lesions
0 score	Normal, clean, thin and transparent.
1 score	Slightly thickened and slightly turbid, or individual local white exudate.
2 score	Grayish white exudate in a few areas of the air sac, moderate sac thickness.
3 score	Majority of the air sacs are fully covered with yellow white caseous exudate and thickening of air sacs is obvious.
4 score	Serious air sac lesions with white thick exudate on thoracic cavity and abdominal cavity.

**Table 2:** Lung lesion scoring in chickens.

Assigned score	Lung lesions
0 score	None.
1 score	Slight oedema of the alveolar walls.
2 score	Moderate oedematous thickening of alveolar walls with occasional alveoli containing coagulated oedema fluids.
3 score	Extensive occurrence of alveolar and interstitial oedema.

### Flow cytometric analysis of T-lymphocyte subsets

The splenic lymphocytes were collected for the measurement of the CD4+/CD8+ ratio as previously described^27^. Briefly, 1 × 10^6^ splenic lymphocytes were incubated with anti-chicken CD3-SPRD, CD4-FITC and CD8-RPE (Southern Biotech, USA) at 4 °C for 30 min. Subsequently, lymphocytes were washed 3 times with phosphate buffer saline containing 1% fetal bovine serum, then resuspended and analyzed by FacsCalibur and CellQuest software (Becton Dickinson, USA). Viable lymphocytes were calculated based on light scatter (forward and side scatter) characteristics, and 10,000 events were analyzed for positive staining with SPRD, FITC and RPE antibodies.

### Statistical analysis

Data were analyzed using IBM SPSS Statistics 22 statistical software, and results were expressed as means ± SD. One-way ANOVA, and Duncan multiple comparison and two-tailed independent Student *t* tests were performed for comparison between groups. Multiple group analysis included the multiple comparison correction (Bonferroni) was conducted. Differences between groups with *P* values of <0.05 or <0.01 were considered to be statistically significant.

## Results

### Classification of *Aspergillus* spp. in poultry feeds

**Figure 1:**
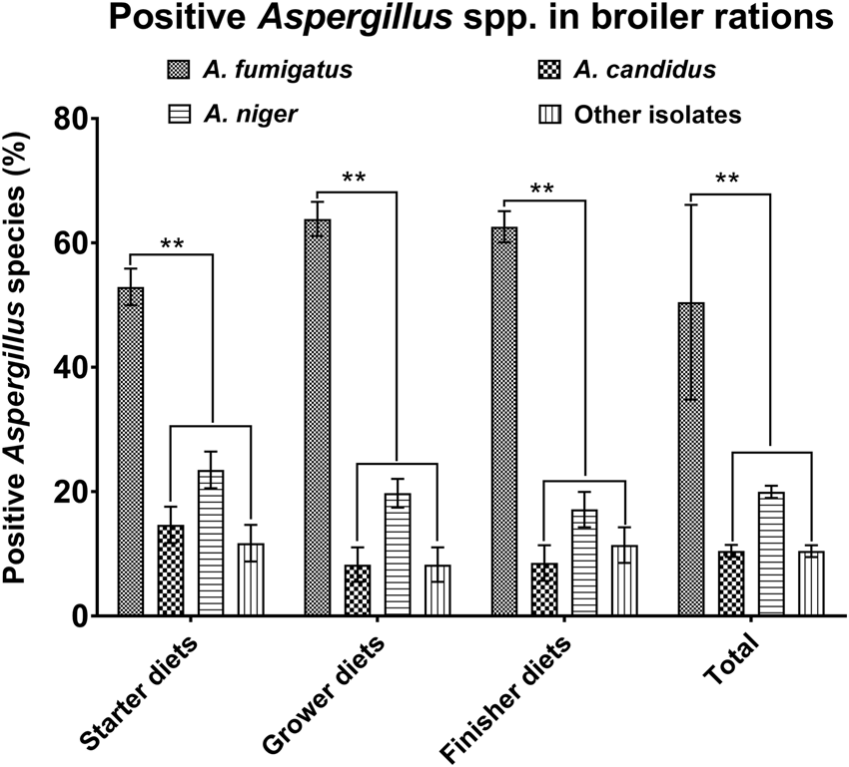
Classification of *Aspergillus* spp. in poultry feeds *A. fumigatus* isolates were dominant specie among *A. candidus*, *A. fumigatus*, *A. niger* and other isolates in three rations. There was a substantial difference between *A. fumigatus* and other pathogens (^**^ *P* < *0.01*). However, no statistical difference was detected between *A. candidus* and *A. niger*.

Of 105 feed samples, 63 (60.0%) were positive for *A. fumigatus*, 21 (20.0%) were positive for *A. niger* and 11 (10.5%) were positive for *A. candidus*. However, 11 samples out of 105 feeds (10.4%) were positive for other isolates (Fig. 1). Obviously, *A*. *fumigatus* isolates accounted for significant statistical differences as compared to other *Aspergillus* species *(P* < *0.01)*. Interestingly, higher *A. fumigatus* conidia were determined both in the grower and finisher diets in comparison with those of the starter diets (*P* < *0.05*).

### Isolation and identification of *Aspergillus fumigatus*

**Figure 2:**
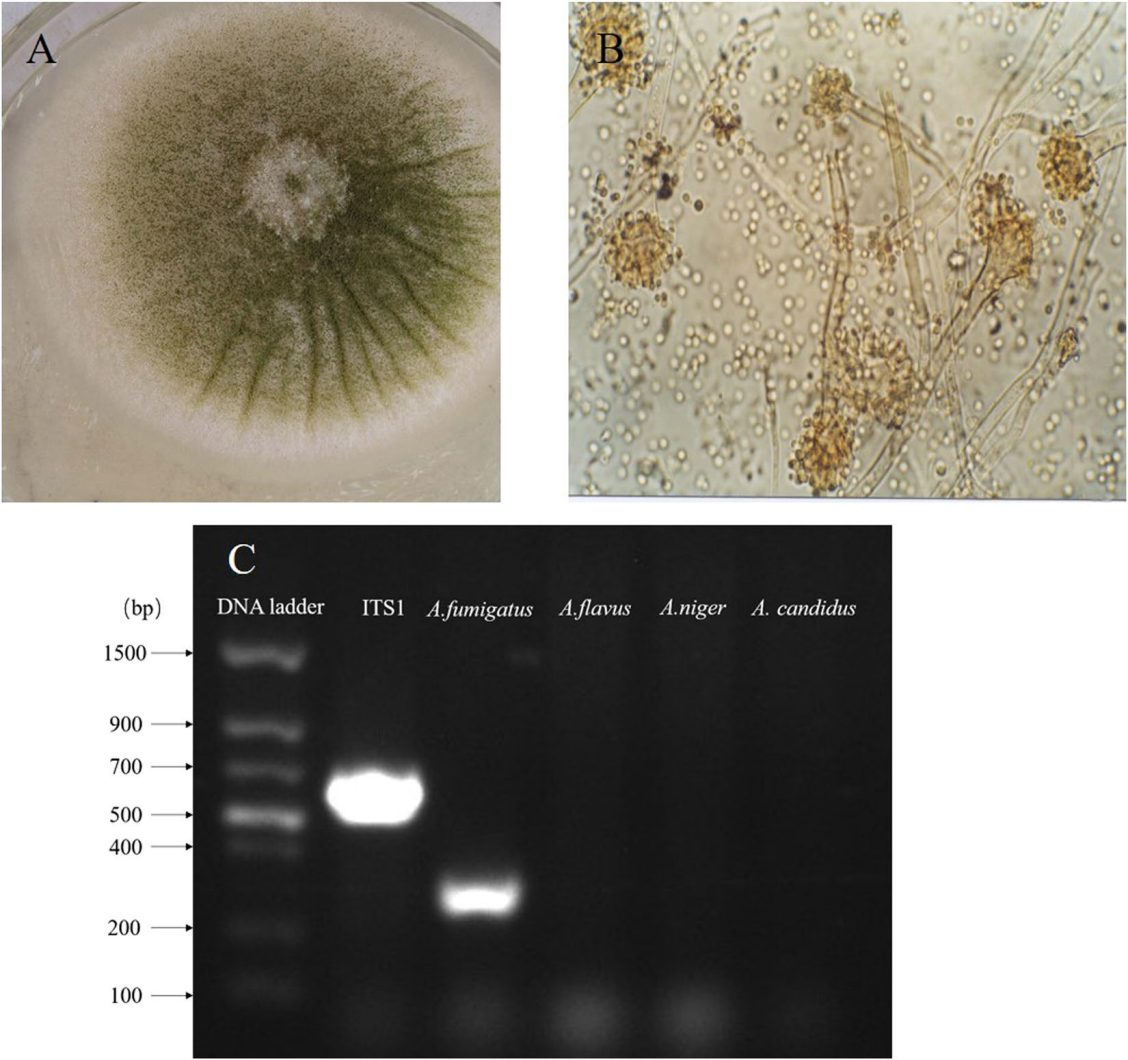
Isolation and identification of *Aspergillus fumigatus (* **A**). The isolation of *A. fumigatus* strains from the feed samples by single colony purification. The colonies of fungus grew rapidly from the broiler’s grower ration. (**B**) Microscopic detection of *A. fumigatus* in broiler rations. The colony is villous, with dark green, green, pale or pale yellow. The conidia have a short columnar head, the conidia wall is smooth, the top sac is flask, and the monospore is in the upper part of the top capsule. (**C**) Identification of *A. fumigatus* by conventional PCR. The product of ITS1 region and the specificity of putatively species-specific primer-sets for *A. fumigatus* were successful amplified.

Mycological examination of the 105 samples indicated that the fungus grew rapidly at 25 °C, and the texture of colonies varied from wooly to cottony, to somewhat granular with smoky gray color. However, the color of very mature colonies turned to slate gray. The surface color was smoky gray at the beginning and became slate gray by aging (Fig. 2A).

Under microscopy, conidial heads were in the form of compact columns in an undisturbed culture. Conidiophores had smooth-walled, greenish, up to 300 µm long, and terminated in a dome (Fig. 2B). Based on general morphology, cultural and molecular characteristics, fungal spores isolated from grower diets were identified as *A. fumigatus* and amounted to 1.1 × 10^4^ CFU/g, while the spores from the starter and finisher diets were 4.0 × 10^6^ CFU/g and 1.0 × 10^5^CFU/g, respectively. The putative *Aspergillus*-specific primers were employed to identify *Aspergillus* spp. As we speculated, the product of 521 bp in length, including the whole ITS1 region was amplified by PCR from all tested samples. Furthermore, species-specific primer-sets for *A. fumigatus*, *A. flavus*, *A. niger* and *A.candidus* were tested. *A. fumigatus* was successfully identified by PCR reactions (Fig. 2C). Finally, after PCR identification, the *A*. *fumigatus* strains were successfully isolated from the feed samples by single colony purification out of *Aspergillus* spp. isolates.

### Effect of co-infection on relative weight gain

**Figure 3:**
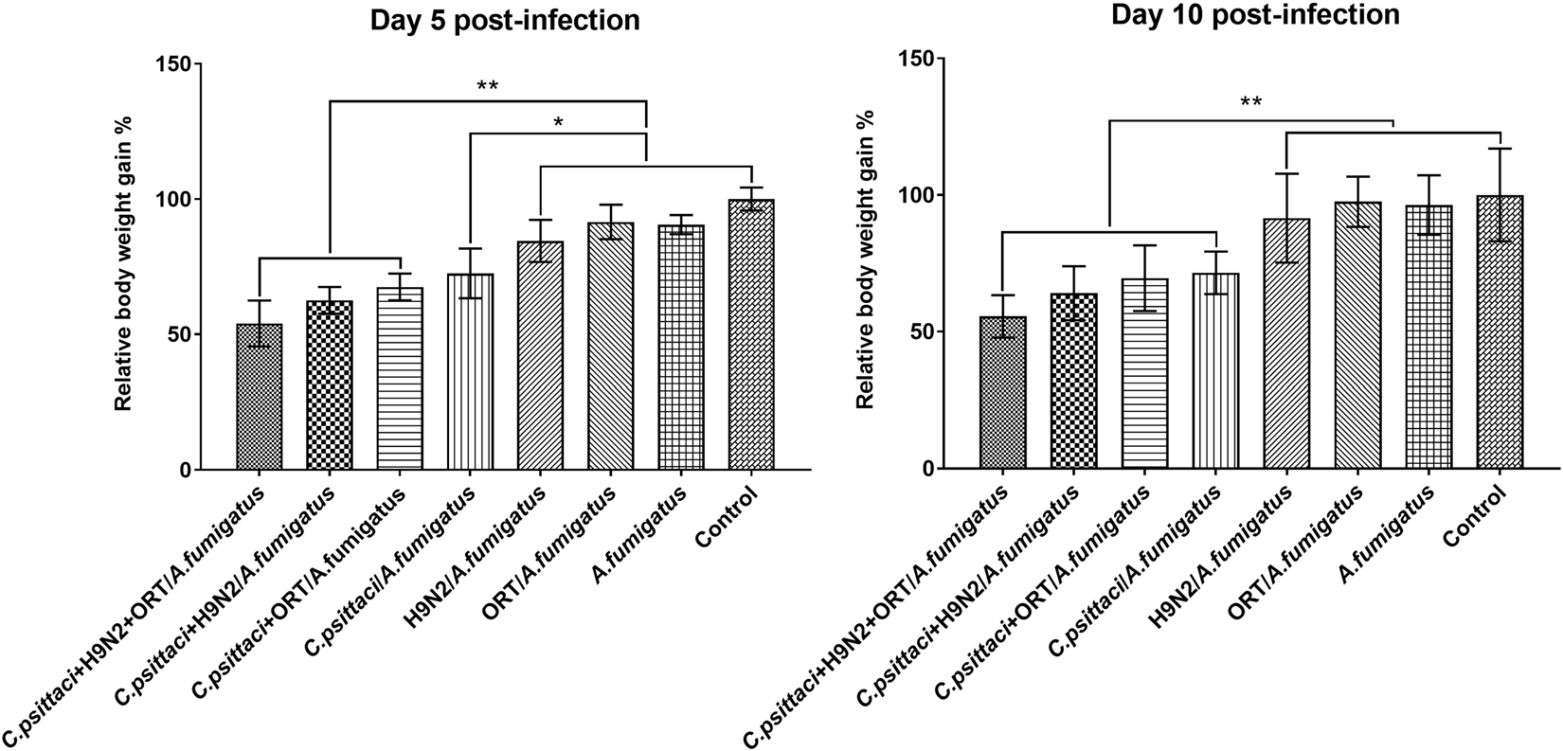
Relative body weight gain post-inoculation. The relative body weight gain in the chickens inoculated with 3 pathogens were highly significant decrease  compared to the birds received H9N2/*A. fumigatus*, ORT/*A. fumigatus*, or *A. fumigatus* group on day 5 p.i. (^**^ *P* < *0.01*) Similarly, chickens infected with *C. psittaci* + H9N2 + ORT/*A. fumigatus*, *C. psittaci* + H9N2/*A. fumigatus*, *C. psittaci* + ORT/*A. fumigatus* and *C. psittaci*/*A. fumigatus* groups were significantly different compared to the H9N2/*A. fumigatus* or ORT/*A. fumigatus* group on day 10 p.i. (^**^ *P* < *0.01*). However, chickens inoculated with *C. psittaci*/*A. fumigatus* were slightly significant decrease compared to the birds inoculated with H9N2/*A. fumigatus*, ORT/*A. fumigatus* or *A. fumigatus* group on day 5 p.i. (*P < *0.05*).

Within five days post infection (p.i.) with *C. psittaci*, H9N2, ORT and *A. fumigatus*, chickens exhibited a decreased appetite and poor activity compared to the healthy control group. The relative body weight gains in those animals infected with several pathogens were 66.4%, 72.2%, and 83.9% in the *C. psittaci* + H9N2 + ORT/*A. fumigatus*, *C. psittaci* + H9N2/*A. fumigatus* and *C. psittaci* + ORT/*A. fumigatus* groups, respectively. However, the relative weight gains were 82.7%, 85.4%, 96.4% and 88.6% in the *C. psittaci*/*A. fumigatus*, H9N2/*A. fumigatus* and ORT/*A. fumigatus* groups, correspondingly. Interestingly, birds infected with the *C. psittaci* + H9N2 + ORT/*A. fumigatus* or *C. psittaci* + H9N2/*A. fumigatus* exhibited a significant lower relative body weight gain as compared to those of the *ORT*/*A. fumigatus* or healthy control group (*P* *<* *0.01*) (Fig. 3).

### Morbidity and mortality post-infection with *C.psittaci*, H9N2, ORT and *A. fumigatus*

**Figure 4:**
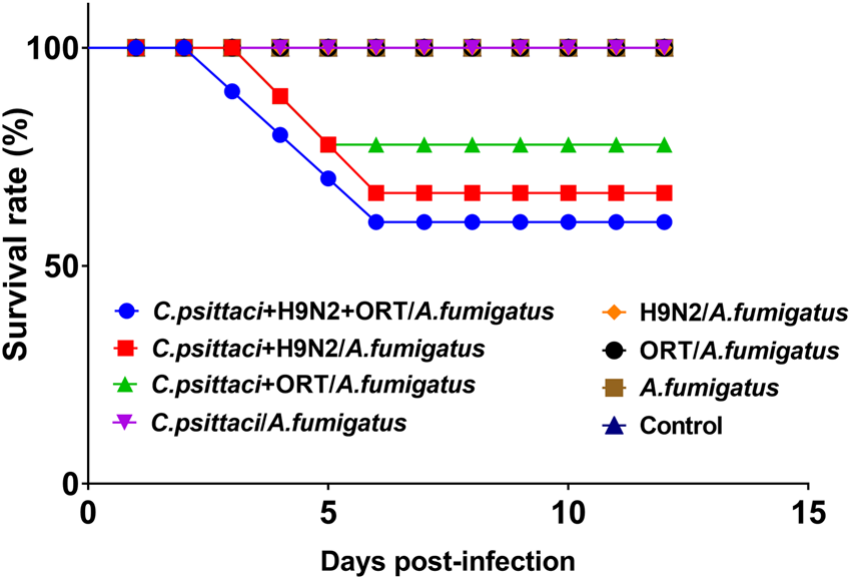
Survival rates in the chicken inoculated with different pathogenic combinations Birds displayed 60.0% survival rates in the *C. psittaci* + H9N2 + ORT/*A. fumigatus* group as compared to 70% live birds in the *C. psittaci* + H9N2/*A. fumigatus* group and 80.0% live birds in the *C. psittaci* + ORT/*A. fumigatus* group, respectively. Statistical differences were found in the three and four infectious agent groups compared to the one or two infectious agent groups as well as the control group (^**^ *P* < *0.01*).

In addition to the poor activity in the birds infected with *C. psittaci*, ORT, H9N2 and the fungal spores, 8 out of 10 chickens (80.0%) suffered from severe breathing difficulties. Subsequently, 4 birds died and the mortality rate was approximately 40%. Meanwhile, of the chickens inoculated with *C. psittaci*, H9N2 and *A. fumigatus* conidia, 5 birds (50%) suffered from typical respiratory distress on day 6 p.i., and the mortality rate was nearly 30.0%. Moreover, 2 chickens out of 10 (20.0%) were died due to the combined infection with *C. psittaci*, ORT and *A. fumigatus* conidia. In general, the birds infected with two pathogens as well as *A. fumigatus* exhibited typical pneumonia, decreased activity, and recovered thereafter. Intriguingly, transient breathing difficulty, decreased activity and appetite with no deaths were recorded in the *C. psittaci* **/** *A. fumigatus*, H9N2/*A. fumigatus* or *A. fumigatus* group during the observation period (Fig. 4).

### Effect of co-infection on lesions of air-sacs and lungs

**Figure 5:**
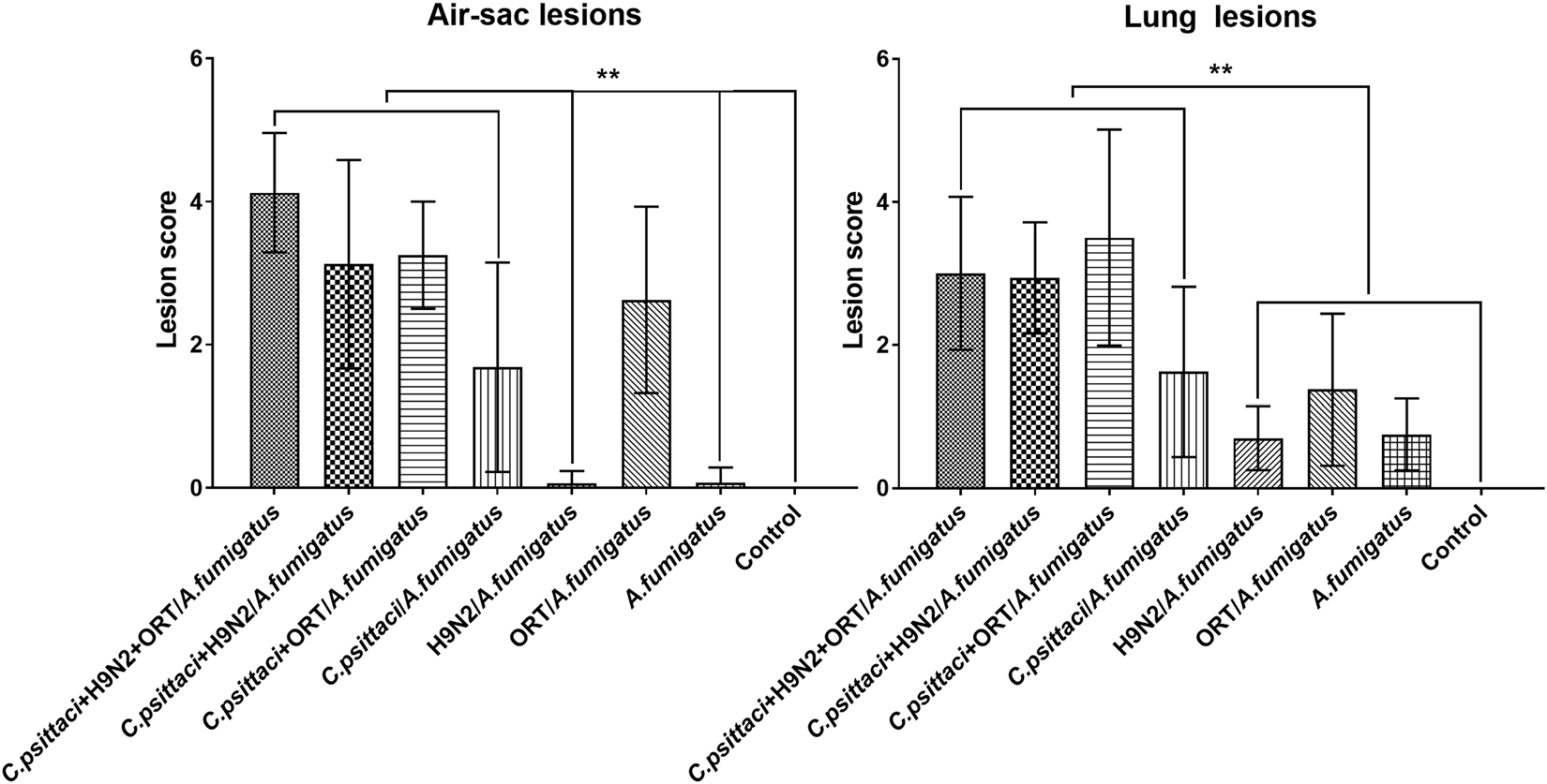
Air-sac and lung lesions in the chickens inoculated with different infectious agent combinations The air-sac lesions of chickens inoculated with *C. psittaci* + H9N2 + ORT/*A. fumigatus*, *C. psittaci* + H9N2/*A. fumigatus*, *C. psittaci* + ORT/*A. fumigatus* or *C. psittaci*/*A. fumigatus* group were markedly different from those received H9N2/*A. fumigatus*, *A. fumigatus* or control group (^**^ *P* < *0.01*). However, the lung lesions of chickens inoculated with *C. psittaci* + H9N2 + ORT/*A. fumigatus*, *C. psittaci* + H9N2/*A. fumigatus*, *C. psittaci* + ORT/*A. fumigatus* or *C. psittaci*/*A. fumigatus* group were significantly different from those received H9N2/*A. fumigatus*, ORT/*A. fumigatus*, *A. fumigatus* or control group (^**^ *P* < *0.01*).

At necropsy, severe hemorrhagic discharges in the tracheal and bronchial obstruction were observed in the *C. psittaci* + H9N2 + ORT/*A. fumigatus*, *C. psittaci* + H9N2/*A. fumigatus* or *C. psittaci* + ORT/*A. fumigatus* group in comparison with the *C. psittaci*/*A. fumigatus*, *ORT*/*A. fumigatus* or H9N2/*A. fumigatus* group. Moreover, fibrinous airsacculitis, pericarditis, peritonitis and haemorrhagic pneumonia were evident in the chickens co-infected with 3 or 4 microbial isolates. As for lesion score of air sacs, chickens inoculated with *C. psittaci* + H9N2 + ORT/*A. fumigatus* exhibited higher lesion score compared to *C. psittaci*/*A. fumigatus* or ORT/*A. fumigatus* on day 10 p.i. (*P* *<* *0.01*). However, the H9N2/*A. fumigatus* and *A. fumigatus* groups displayed the lowest air-sac lesion scores compared to other groups (Fig. 5).

With respect to lung lesions, severe haemorrage and necrosis were noted in the *C. psittaci* + H9N2 + ORT/*A. fumigatus*, *C. psittaci* + H9N2/*A. fumigatus* or *C. psittaci* + ORT/*A. fumigatus* group when compared to those in the ORT/*A. fumigatus* or *C. psittaci/A. fumigatus* group (*P* < *0.01*). However, lower lung lesions were observed in the *A. fumigatus* and H9N2/*A. fumigatus* groups (Fig. 5).

### Effect of the co-infection on immune organ index

As for the immune organ indices of the chickens with the above 3 pathogen inoculations, both spleen and bursa indices were substantially elevated compared to those with ORT/*A. fumigatus* or *A. fumigatus* alone.

**Figure 6:**
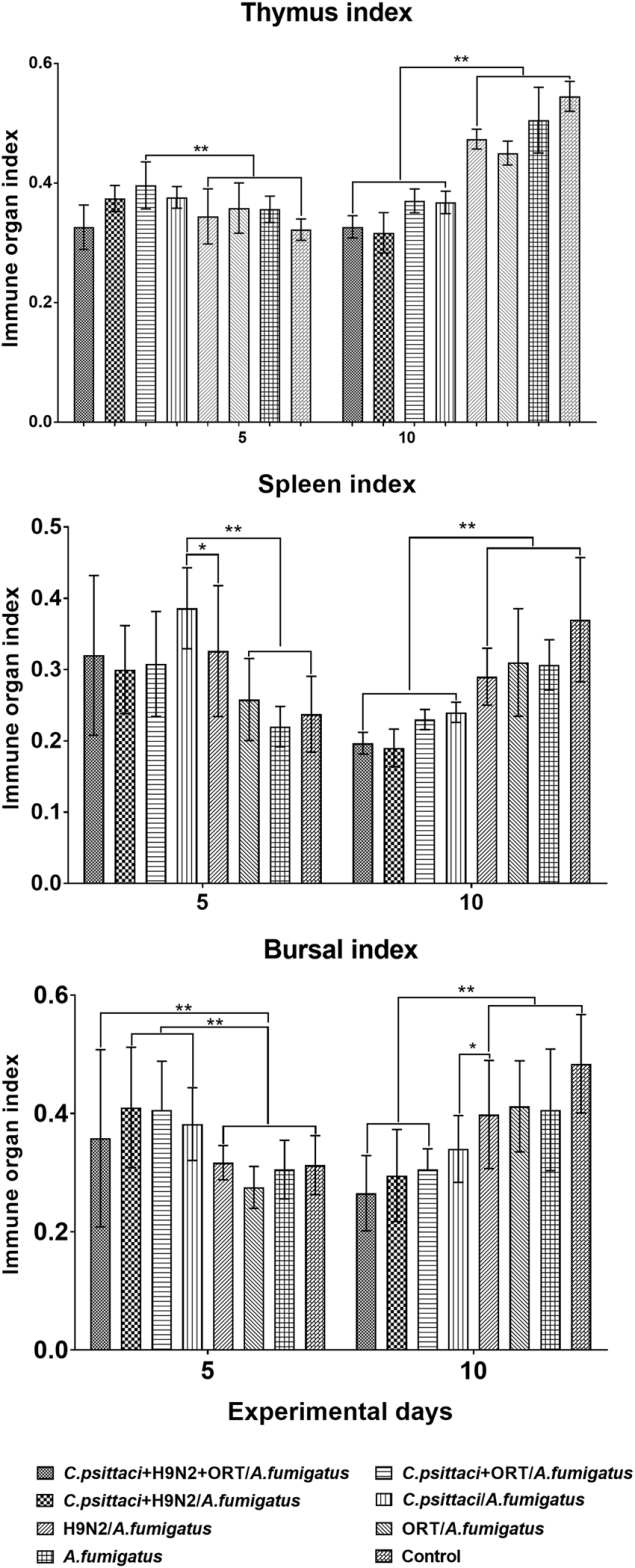
Immune organ indices in the chicken inoculated with different pathogenic combinations. The thymus index of birds inoculated with the *C. psittaci* + ORT/*A. fumigatus* group was substantially different compared to the H9N2/*A. fumigatus* or control group (**P < *0.01*). However, no significant difference was found among the *C. psittaci* + H9N2 + ORT/*A. fumigatus*, *C. psittaci* + H9N2/*A. fumigatus* and *C. psittaci* + ORT/*A. fumigatus* groups on the same time points. Furthermore, the spleen index of chickens infected with the *C. psittaci*/*A. fumigatus* group was substantially different as compared to the H9N2/*A. fumigatus* (*P < *0.05*), ORT/*A. fumigatus*, *A. fumigatus* or control (**P < *0.01*) group on day 5 p.i. On day 10, a significant decrease was recorded in the *C. psittaci* + H9N2 + ORT/*A. fumigatus*, *C. psittaci* + ORT/*A. fumigatus*, *C. psittaci* + H9N2/*A. fumigatus* or *C. psittaci*/*A. fumigatus* groups in comparison with the H9N2/*A. fumigatus* or control group (**P < *0.01*). Regarding the bursal index, a significant decrease was obvious in the *C. psittaci* + H9N2 + ORT/*A. fumigatus*, *C. psittaci* + ORT/*A. fumigatus* or *C. psittaci* + H9N2/*A. fumigatus* group in comparison with the H9N2/*A. fumigatus* or control group (**P < *0.01*) on day 10 p.i.

Lately, the spleen, thymus as well as bursal indices were significantly reduced in the *C. psittaci* + H9N2 + ORT/*A. fumigatus*, *C. psittaci* + H9N2/*A. fumigatus* or *C. psittaci*/*A. fumigatus* group as compared to those of the H9N2/*A. fumigatus* or ORT/*A. fumigatus* group (*P* < *0.01*). On day 10, the bursal index of *C. psittaci*/*A. fumigatus* group was lower than the H9N2/*A. fumigatus* or control group (*P* < *0.05*). However, there were no significant differences in the immune organ index between the *C. psittaci* + H9N2 + ORT/*A. fumigatus* and *C. psittaci* + H9N2/*A. fumigatus* groups (Fig. 6).

### Effect of co-infection on subsets of spleen lymphocytes

**Figure 7:**
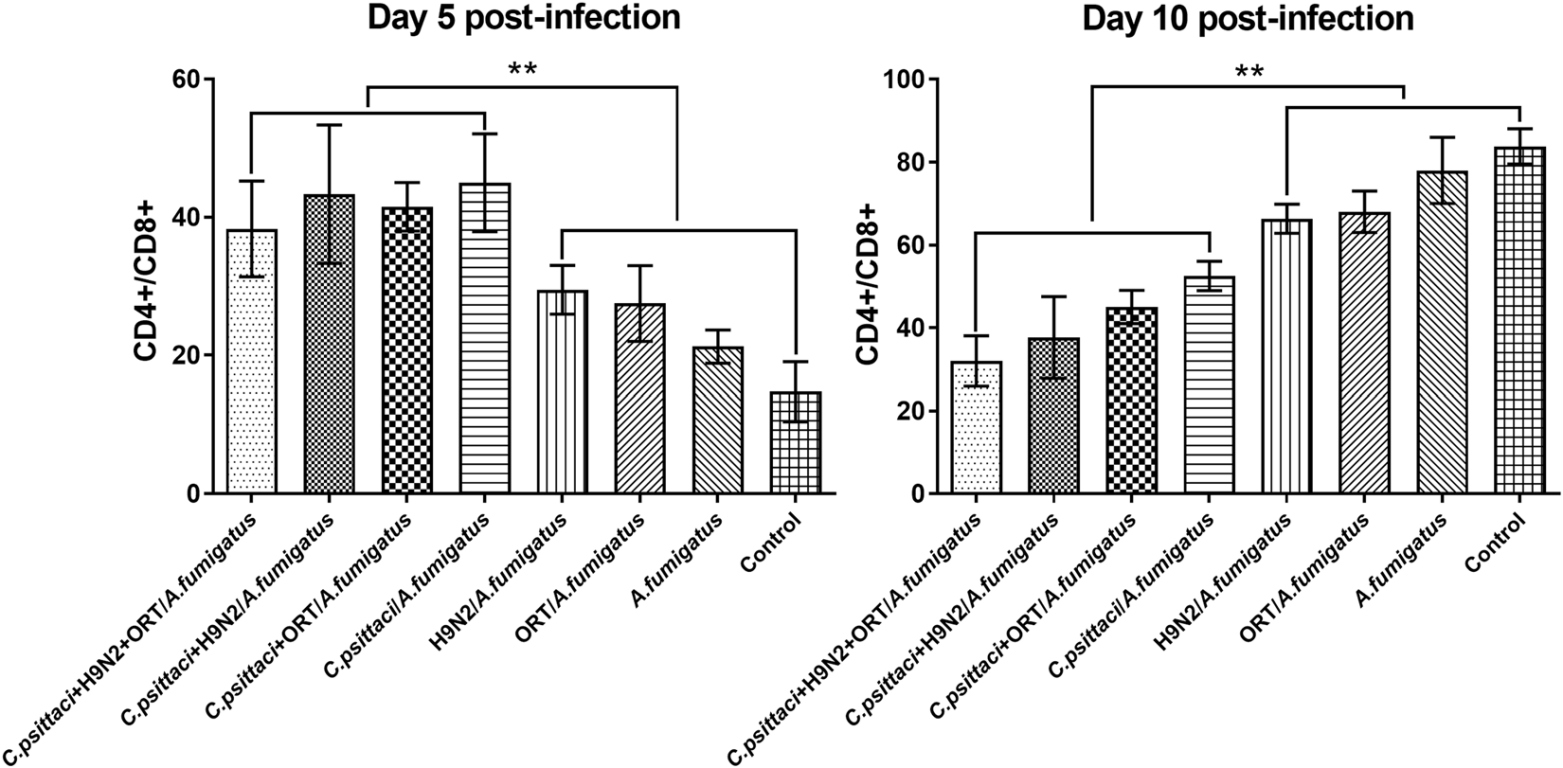
Splenic lymphocytes ratio of CD4+/CD8+ in the chickens inoculated with diverse infectious agent combinations There was a significant increase in the ratio of splenic lymphocytes, CD4+/CD8+ (**P < *0.01*) in the birds inoculated with the *C. psittaci* + H9N2 + ORT/*A. fumigatus*, *C. psittaci* + ORT/*A. fumigatus*, *C. psittaci* + H9N2/*A. fumigatus* or *C. psittaci*/*A. fumigatus* group compared to the chickens received the H9N2*/A. fumigatus*, ORT/*A. fumigatus* or *A. fumigatus* group on day 5 p.i. However, a significant decrease in the ratio of CD4+/CD8+ (**P < *0.01*) when the chickens infected with *C. psittaci* + H9N2 + ORT/*A. fumigatus*, *C. psittaci* + ORT/*A. fumigatus* and *C. psittaci* + H9N2/*A. fumigatus* or *C. psittaci*/*A. fumigatus* group compared to the birds infected with H9N2*/A. fumigatus*, ORT/*A. fumigatus*, *A. fumigatus* or control group.

At day 5 post-inoculation, a significantly higher CD4+/CD8+ ratio was detected in the *C. psittaci* + H9N2 + ORT/*A. fumigatus* or *C. psittaci* + H9N2/*A. fumigatus* group when compared to that of the ORT*/A. fumigatus* or H9N2/*A. fumigatus* group (*P* < *0.01*). Additionally, the ratio of CD4+/CD8+ was significantly reduced in the *C. psittaci* + H9N2/*A. fumigatus* and *C. psittaci*/*A. fumigatus* groups compared to that of the H9N2/*A. fumigatus* or *A. fumigatus* group (Fig. 7).

## Discussion

Our survey of chickens in the diseased farms indicated that a highly contamination of *Aspergillus* spp. was prevalent in the chicken rations. Of the rations sampled, 60.0%, 20% and 10.5% were positive for *A. fumigatus*, *A. niger* and *A. candidus*, respectively while *A. fumigatus* spores were highly reported both in the grower and finisher rations, suggesting a severe fungus-elicited high risk to the poultry health as well as a potential food safety problem for human beings. Interestingly, the dominant *A. fumigatus* might pose an immune suppression and a secondary microbial infection. Both a significant decrease in relative weight gain and a severe airsacculitis with 40% mortality rate were replicated in the birds inoculated with *C. psittaci* + H9N2 + ORT*/A. fumigatus*. Similarly, post-inoculation with 2 or 3 pathogens in SPF chickens could lead to the same symptoms with a milder degree. The respiratory signs and mortality rates were characterized as those seen in the clinical outbreak of seasonal avian pneumonia in broiler poultry under cold temperature. No deaths occurred in the birds infected with *C. psittaci*/*A. fumigatus*, H9N2/*A. fumigatus* and *A. fumigatus* conidia alone, and these chickens only suffered from transient respiratory distresses. In a previous record, no clinical signs and mortality rates were noted in SPF chickens whereas more than 50% of mortality rate was occurred in broilers by aerosol inoculation with *A. fumigatus* ^28^. The results of the present study might suggest that co-infection with *C. psittaci*, H9N2, ORT and *A. fumigatus* are responsible for the current severe airsacculitis with high mortality rates in commercial broilers. It is the first time that clinical airsacculitis has been well replicated using the above clinical four pathogenic isolates in a SPF chicken model. A post-mortem examination implied that *C. psittaci* and ORT infection targeted both air sacs and lungs while H9N2 infection induced upper airway lesions at the junctions of the primary and secondary bronchi and lungs in comparison with the lung damage caused by an *A. fumigatus* infection. Based on the dominant lesions in the co-infection, *C. psittaci* infection might worsen avian airsacculitis by inducing immune suppression characterized by the decreased immune organ indices and ultimately lowered CD4+/CD8+ ratios^14^. After replacement of the grower ration with *A. fumigatus-* contaminated ration, the immune suppression was aggravated by the *C. pisttaci*, H9N2 and ORT infection, contributing to the 40% mortality rate. Relatedly, 10%-30% mortality rate was reported in broilers with *C. psittaci* infection^29^. Moreover, the birds developed upper respiratory disease and dyspnoea, and *C. psittaci* infection preceded the ORT infection^9^. During acute *C. psittaci* infection, aMPV infection might play a role in exacerbating the respiratory disease. However, no clear interaction could be established after aMPV infection in latently *C. psittaci-* infected SPF turkeys^30^. In our pilot survey, an average of 68.4% *C. psittaci*-specific positive antibodies were detected in the affected chickens, suggesting that highly infection with *C. psittaci* might contribute to an avian respiratory disease. In recent study, birds inoculated with *C. psittaci*/H9N2 induced 37.5% mortality with typical breath difficulty, and immune suppression was identified in virulent *C. psittaci*- infected SPF chickens^14^.

In comparison with *C. psittaci* infection, *A. fumigatus* contamination is often trivial in the co-infection, particularly at the level of 1.1 × 10^4^ CFU/g in broiler rations, and more than 2.3 × 10^4^ CFU/g in turkey commercial feed^31^. *A. fumigatus* is the predominant mold pathogen of the immunosuppressed bird, characterized by reducing NDV antibody, phagocytic activity and reactive oxygen species (ROS) production^19^. Once the immune system is compromised, the conidia of *A. fumigatus* can germinate into hyphae and establish a focus of infection within the lung. In addition to *A. fumigatus* contamination, aflatoxins (AF) produced by *Aspergillus flavus* and *Aspergillus parasiticus* are common contaminants of feed raw materials^32^ and previous studies have also reported the presence of aflatoxins in poultry tissues in Pakistan^33,34^. In our survey, *A. flavus* and *A. parasiticus* were isolated much less frequently than *A. fumigatus* from the chicken ration samples. Therefore, a study of the association between mycotoxin and feed-borne *A. fumigatus* is urgently needed.

Clinically, outbreaks of avian airsacculitis occur frequently in poultry flocks even after immunization with many vaccines, including inactivated H9N2 vaccine, attenuated MG, live IBV and NDV vaccine. This dilemma suggests the involvement of other pathogens during respiratory diseases. Our study implies that multiple infectious agents may contribute to respiratory diseases. We propose that *C. psittaci* triggers primary immune suppression and aggravates the compromised birds by the consistent feed-borne *A. fumigatus*, contributing to the secondary infection with H9N2 and ORT and severe respiratory distress with high mortality rates.

Our study is the first to report typical airsacculitis with a 40% mortality rate in SPF chickens infected with *C. psittaci*, ORT, AIV H9N2 and *A. fumigatus*. During co-infection, both *C. psittaci* and *A. fumigatus* induce suppression by the impairment of immune organs and Th1/Th2 imbalance, while the combination of H9N2 with ORT might play an exacerbating role in the respiratory disease by causing lung damage. Therefore, it is urgent to eradicate immune suppression caused by *C. psittaci* infection and feed-borne *A. fumigatus*. Furthermore, the combination of *C. psittaci*, ORT, H9N2 and *A. fumigatus* infection should be considered when developing prevention and treatment strategies for avian airsacculitis.
